# Validation of clinic-based cryptococcal antigen lateral flow assay screening in HIV-infected adults in South Africa

**DOI:** 10.1038/s41598-018-37478-7

**Published:** 2019-02-25

**Authors:** Paul K. Drain, Ting Hong, Meighan Krows, Sabina Govere, Hilary Thulare, Carole L. Wallis, Bernadett I. Gosnell, Mahomed-Yunus Moosa, Ingrid V. Bassett, Connie Celum

**Affiliations:** 1 Departments of Global Health, University of Washington, Seattle, USA; 2 Medicine, University of Washington, Seattle, USA; 3 Epidemiology, University of Washington, Seattle, USA; 4 AIDS Healthcare Foundation, Durban, South Africa; 5 BARC-SA and Lancet Laboratory, Johannesburg, South Africa; 6 Department of Infectious Diseases, University of KwaZulu-Natal, Durban, South Africa; 7 Department of Medicine, Massachusetts General Hospital, Harvard Medical School, Boston, USA

## Abstract

Since rapid cryptococcal antigen lateral flow assays (CrAg LFA) may expedite treatment of HIV-associated cryptococcal infections, we sought to validate clinic-based CrAg LFA testing. Among newly-diagnosed HIV-infected adults in South Africa, a trained nurse performed clinic-based testing of urine, fingerprick capillary and venous whole blood with rapid CrAg LFA (Immy Diagnostics, Norman, USA). We performed matched laboratory-based serum cryptococcal antigen testing with an enzyme immunoassay (EIA). We assessed diagnostic accuracy using EIA as the gold-standard, and performed additional validation testing on serum and among hospitalized adults with cryptococcal meningitis. Among 5,618 participants enrolled, 1,296 were HIV-infected and screened for cryptococcal antigenemia. Overall CrAg prevalence by serum EIA was 3.6% (95% CI 2.0–6.0%) for adults with CD4 < 200 cells/mm^3^, and 5.7% (95% CI 2.8–10.2%) for adults with CD4 < 100 cells/mm^3^. Using expanded screening guidelines (CD4 < 200 cells/mm^3^), CrAg LFA testing of venous whole blood, fingerprick capillary blood, and urine had diagnostic sensitivities of 46% (95% CI 19–75%), 38% (95% CI 14–68%), and 54% (95% CI 25–81%), and specificities of 97%, 97%, and 86%, respectively. When tested on serum samples, CrAg LFA had sensitivity of 93% (95% CI 66–100%) and specificity of 100% (95% CI 88–100%). All venous and fingerprick whole blood CrAg LFA tests were positive among 30 hospitalized adults with cryptococcal meningitis. Two independent readers had strong agreement for all LFA results (p < 0.0001). When performed at the point-of-care by trained nurses, CrAg LFA testing was feasible, had the highest accuracy on serum specimens, and may accelerate treatment of HIV-associated cryptococcal infections.

## Introduction

Cryptococcosis is an opportunistic fungal infection that causes approximately 15% of AIDS-related deaths worldwide, the majority of which occur in sub-Saharan Africa^1^. In South Africa, cryptococcal infections accounted for approximately 63% of meningitis cases, due in part to the high burden of HIV/AIDS^2^. Cryptococcal capsular antigens (CrAg) can be detected before the onset of symptomatic cryptococcal meningitis^3–5^, and oral fluconazole can reduce risks of cryptococcal meningitis and mortality^6–9^. The World Health Organization (WHO) recommends CrAg screening for all antiretroviral therapy (ART)-naïve HIV-infected individuals with CD4 T-cell count <100 cells/mm^3^ ^10^, and consideration for CrAg screening for those with CD4 100–199 cells/mm^3^ ^11^.

Several sub-Saharan African countries have incorporated CrAg screening into guidelines, and some are evaluating reflex laboratory-based CrAg testing for blood samples with CD4 <100 cells/mm^3^ ^1^. Since survival benefit is related to early CrAg screening and prompt initiation of pre-emptive fluconazole therapy, minimizing the time from first clinic visit to testing and subsequent treatment are critical^12^. A rapid lateral flow assay (LFA) was developed to expedite CrAg screening and has demonstrated good accuracy on serum and cerebrospinal fluid specimens^13–16^. However, survival benefit may be optimized by integrating early CrAg screening and fluconazole pre-emptive therapy while the fungal burden is relatively low prior to the onset of symptomatic meningitis^7,9,12^.

Delays associated with laboratory-based CrAg screening could be averted if the CrAg LFA is suitable for use at the clinical point of care. Therefore, the objective was to validate clinic-based rapid CrAg LFA testing on venous whole blood, fingerprick capillary blood, and urine among HIV-infected adults before ART initiation in an ambulatory clinic in South Africa, compared to the gold-standard of an EIA with the same antigens performed on serum.

## Methods

### Study design and participants

We enrolled persons who presented for voluntary HIV testing at the iThembalabantu Clinic in the Umlazi township of KwaZulu-Natal, South Africa from September 2013 to April 2017. The clinic tests approximately 150 adults for HIV each month and provides free clinic- and community-based HIV care and treatment for over 10,000 HIV-infected patients. We enrolled English or Zulu speaking adults ≥18 years of age, who presented to the clinic for HIV testing and were willing and able to provide written informed consent for study participation. We excluded pregnant females and those taking anti-fungal therapy in the prior three months. The study was approved by the University of Washington’s Institutional Review Board (IRB #49563) and the University of KwaZulu-Natal’s Medical Research Ethics Committee (Protocol #BF052/13). In addition, all experiments were performed in accordance with relevant guidelines and regulations.

### Data collection

Research assistants completed a sociodemographic questionnaire, and HIV counselors performed serial rapid HIV testing according to South African guidelines^17^. Among HIV-infected participants, research nurses obtained a medical history, administered a clinical symptom questionnaire, and requested blood and urine samples for clinic- and laboratory-based testing, including CD4 T-cell count using FACSCalibur™ (BD, San Jose, CA). All participants received routine medical care according to local guidelines^17^.

### Clinic-based cryptococcal antigen testing

Trained research nurses conducted clinic-based CrAg LFA tests (Immy Diagnostics, Norman, Oklahoma, USA), according to the manufacturer’s instructions, on venous whole blood, fingerprick capillary blood, and urine samples from each participant. First, capillary blood was obtained by pricking the sterilized pad of a forefinger and transferring whole blood directly onto an LFA test strip. Second, 40 μL of venous whole blood was obtain from a 4 ml EDTA vial of blood drawn from an antecubital vein using a sterile pipette. Third, 40 μL of urine was obtained using a sterile pipette from a participant’s urine specimen.

A research nurse then placed each CrAg LFA test strip in a 1.5-mL Eppendorf tube containing 2 drops of “specimen diluent” for the blood tests, or “urine diluent” for the urine test, provided by the test manufacturer. All tests rested upright at room temperature before interpretation at 10 minutes. All tests were independently read by two trained readers, and positive tests were scored from 1 + (low) to 5 + (high) using the manufacturer’s reference. In addition, we performed positive control testing with a CrAg-spiked solution provided by the test manufacturer and according to their instructions, and these spiked tests were consistently positive.

### Laboratory-based cryptococcal testing

Antecubital venous blood specimens were collected at the same time the clinic-based specimens were obtained, and transported to the laboratory for separation into plasma and serum before freezing. The BARC Lab (Johannesburg, South Africa) performed testing on thawed serum using the ALPHA Cryptococcal Antigen enzyme immunoassay (CrAg EIA) test system developed by Immy Diagnostics (Norman, Oklahoma, USA). This CrAg EIA detects the same capsular polysaccharide antigens as the CrAg LFA by Immy Diagnostics. Briefly, 50 μL of plasma was added to the anti-CrAg microwells and the EIA test was performed according to the manufacturer’s instructions. The optical density (OD) was determined with a microplate reader at 450 nm, and samples with an OD reading greater than 0.265 were resulted as positive, which was specified in the product materials by the assay manufacturer (Immy Diagnostics). All laboratory-based EIA testing was blinded to participants’ clinical information, including CD4 count and CrAg LFA results.

The Global Labs (Durban, South Africa) performed testing on thawed serum for latex agglutination using the Cryptococcal Antigen Latex Agglutination System (CALAS^®^, Meridian Biosciences Inc.), according to the manufacturer’s instructions. In brief, 200 μl of serum was incubated with 200 μl of pronase solution at 56 °C for 15 minutes, before placing the solution in a boiling water bath. Then, 25 μl of the participant specimen was added to rings on the test card, along with one drop of detection latex solution. Each well was mixed and visually assessed for agglutination.

### Validation of CrAg LFA on serum and persons with HIV-associated cryptococcal meningitis

After we obtained the initial validation results, we performed *post hoc* testing using the CrAg LFA on 14 CrAg EIA-positive serum samples and 29 randomly-selected CrAg EIA-negative serum samples from our biorepository. All serum samples were allowed to thaw at room temperature and then tested with the CrAg LFA, in the same manner as above. Each CrAg LFA test on serum was independently read by two readers, who were blind to participant clinical information and prior CrAg EIA result.

To further validate the CrAg LFA and our procedures, we enrolled a subset of hospitalized patients with a known diagnosis of cryptococcal meningitis at either King Edward VIII Hospital or Mahatma Gandhi Memorial Hospital in Durban. Each participant had positive laboratory-based latex agglutination tests by both serum and cerebrospinal fluid, which were performed by the National Health Laboratory Service, during their current hospital admission. Within three days of diagnosing cryptococcal meningitis, we recorded demographic and clinical information, including most recent CD4 cell count, and performed testing using the CrAg LFA on venous whole blood, fingerprick capillary blood, and urine specimens, using the same procedures described above.

### Statistical analyses

The primary outcome was diagnostic accuracy of the CrAg LFA for use on venous whole blood, fingerprick capillary blood, and urine, as compared to a gold-standard laboratory-based CrAg EIA result. For sensitivity analyses, we used a combined reference standard for either a positive CrAg EIA or latex agglutination test result. Diagnostic accuracy was assessed using sensitivity, specificity, positive and negative predictive values, and positive and negative likelihood ratios, using STARD guidelines^18^. We assessed agreement between two independent readers of clinic-based CrAg LFA testing using weighted Kappa statistics. Diagnostic accuracy results were analyzed among all participants and stratified by CD4 < 200 cells/mm^3^ and <100 cells/mm^3^, which are consistent with current and proposed guideline recommendations^10,11^. We calculated 95% confidence intervals (CI) and used SAS 9.4 (Cary, USA).

## Results

**Figure 1:**
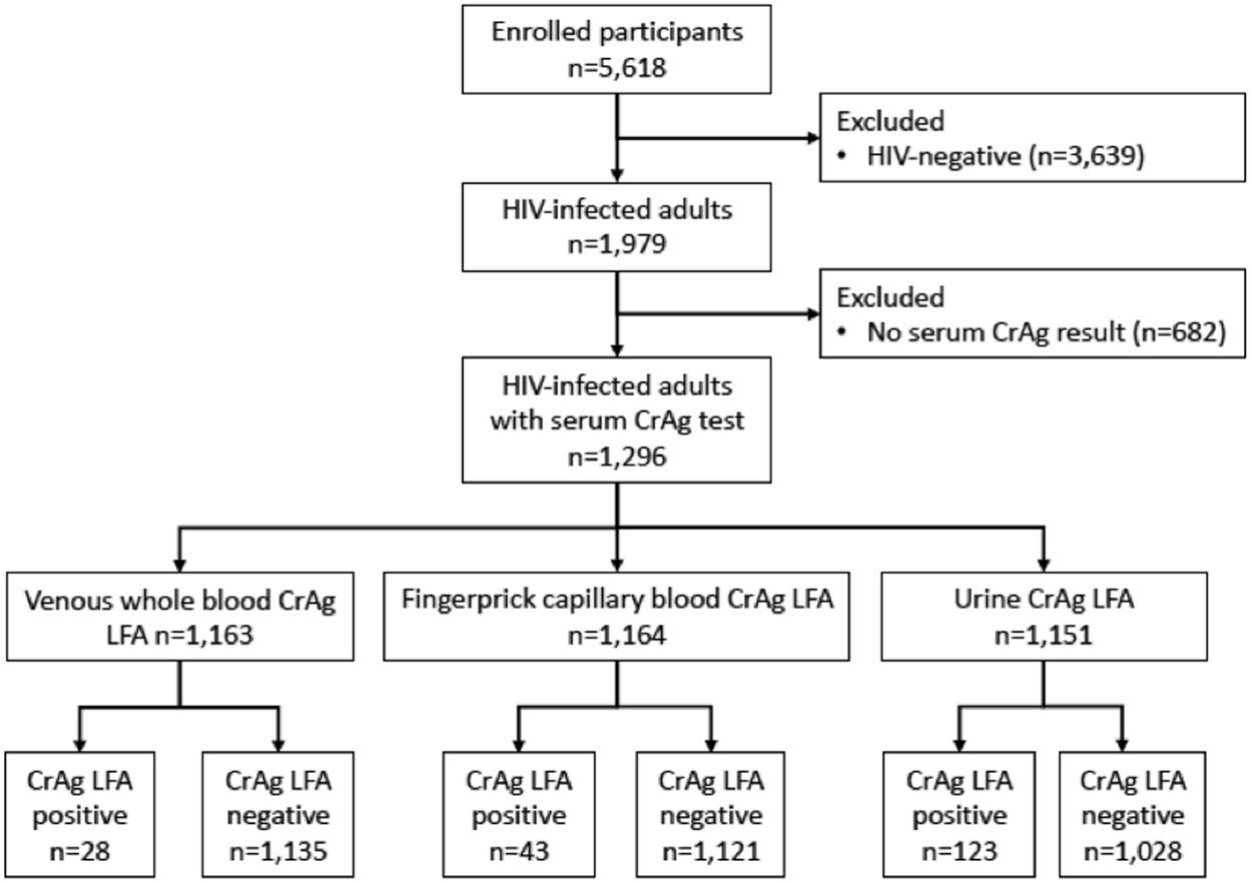
Consort diagram of study enrollment and testing results.

We enrolled 5,618 participants, among whom 1,296 were HIV-infected and had laboratory-based serum CrAg testing by EIA (Fig. 1). Among the HIV-infected participants, mean age was 33.4 (SD ± 9.1) years and 766 (59.1%) were female (Table 1). One participant reported a prior CrAg-positive test, and no participant reported receiving prior treatment for a cryptococcal infection. Participants commonly reported clinical symptoms that have been associated with cryptococcal infection or meningitis, including fatigue (47%), headache for >24 hours (30%), weakness in legs (17%), neck stiffness (15%), weakness in arms (14%), difficulty walking (14%), nausea/vomiting (12%), blurry/double vision (7%), confusion (5%), and seizure within last 7 days (1%). The median CD4 count was 306 cells/mm^3^ (interquartile range: 168–475 cells/mm^3^); 384 (31%) participants had CD4 < 200 cells/mm^3^ and 176 (14%) participants had CD4 < 100 cells/mm^3^.

**Table 1:** Characteristics of the HIV-positive adult study population in Durban, 2013–2017.

	Mean ± SD or N (%) (N = 1,296)
**Sociodemographics**	
Age	33.4 ± 9.1 years
Female	766 (59.1%)
Zulu Ethnicity^a^	726 (93.8%)
**History of HIV and Cryptococcus Testing**	
Previously tested for HIV	813 (62.7%)
Ever tested Cryptococcus positive	1 (0.1%)
**Clinical Symptoms and Examination**	
Fatigue	608 (47.0%)
Headache for >24 hours	388 (29.9%)
Fever	385 (29.7%)
Loss of appetite	323 (24.9%)
Weakness in legs	217 (16.8%)
Neck stiffness	198 (15.3%)
Weakness in arms	186 (14.4%)
Difficulty walking	177 (13.7%)
Nausea or vomiting	151 (11.7%)
Presence of oral thrush	140 (10.9%)
Blurry or double vision	96 (7.4%)
Confusion	58 (4.5%)
Seizure within last 7 days	13 (1.0%)
**CD4 T-cell count**	
Median [IQR] cells/mm^3^	306 [168, 475]
<200 cells/mm^3^	384 (30.5%)
<100 cells/mm^3^	176 (14.0%)

IQR = interquartile range; SD = standard deviation.

^a^522 participants did not have ethnicity recorded.

^*^CrAg LFA tested on 1,163 venous whole blood, 1,164 fingerprick capillary blood, and 1,151 urine specimens.

In this cohort, 15 participants were serum CrAg positive by laboratory-based CrAg EIA testing, and the estimated CrAg prevalence was 1.2% (95% CI 0.6–1.9%; Table 2). Fourteen and 10 of those 15 adults had CD4 < 200 and <100 cells/mm^3^, respectively. The estimated serum CrAg prevalence was 3.6% (95% CI 2.0–6.0%) among participants with CD4 < 200 cells/mm^3^, and 5.7% (95% CI 2.8–10.2%) among participants with CD4 < 100 cells/mm^3^. During clinic-based CrAg LFA testing, 28 (2.4%), 43 (3.7%), and 123 (10.7%) participants were positive by venous whole blood, fingerprick capillary blood, and urine samples, respectively. When stratified by CD4 < 200 cells/mm^3^, CrAg LFA testing of venous whole blood and fingerprick capillary blood had an estimated prevalence of 4.5% (95% CI 2.5–7.3%). Among those with CD4 < 100 cells/mm^3^, estimated CrAg LFA prevalence was 7.1% (95% CI 3.6–12.3%) by venous whole blood, and 5.8% (95% CI 2.7–10.7%) by fingerprick capillary blood.

**Table 2:** Estimated overall and CD4-stratified* cryptococcal antigen (CrAg) prevalence by laboratory-based serum CrAg EIA and clinic-based CrAg LFA testing (N = 1,296).

	#Positive	#Tested	Estimated Prevalence (95% CI)
**Laboratory-based serum CrAg EIA**			
All participants	15	1,296	1.2% (0.6–1.9%)
CD4 ≥200 cells/mm^3^	1	873	0.1% (0.0–0.6%)
CD4 <200 cells/mm^3^	14	384	3.6% (2.0–6.0%)
CD4 <100 cells/mm^3^	10	176	5.7% (2.8–10.2%)
**Clinic-based venous whole blood CrAg LFA**			
All participants	28	1,163	2.4% (1.6–3.5%)
CD4 ≥200 cells/mm^3^	12	790	1.5% (0.8–2.6%)
CD4 <200 cells/mm^3^	15	336	4.5% (2.5–7.3%)
CD4 <100 cells/mm^3^	11	156	7.1% (3.6–12.3%)
**Clinic-based fingerprick capillary blood CrAg LFA**			
All participants	43	1,164	3.7% (2.7–4.9%)
CD4 ≥200 cells/mm^3^	27	792	3.4% (2.3–4.9%)
CD4 <200 cells/mm^3^	15	335	4.5% (2.5–7.3%)
CD4 <100 cells/mm^3^	9	156	5.8% (2.7–10.7%)
**Clinic-based urine CrAg LFA**			
All participants	123	1,151	10.7% (9.0–12.6%)
CD4 ≥200 cells/mm^3^	64	783	8.2% (6.4–10.3%)
CD4 <200 cells/mm^3^	53	331	16.0% (12.2–20.4%)
CD4 <100 cells/mm^3^	33	153	21.6% (15.3–28.9%)

CI = confidence interval; CrAg = cryptococcal antigen; EIA = enzyme immunoassay; LFA = lateral flow assay.

^*^39 participants did not have a CD4 T-cell count result.

The overall agreement between two readers of the CrAg LFA results for positive versus negative was very high for venous whole blood (κ = 0.98), fingerprick capillary blood (κ = 0.97), and urine (κ = 1.0). There was also strong agreement on the score for positive urine CrAg LFA results (weighted κ = 0.85; 95% CI 0.75–0.94), and very high agreement for positive venous whole blood (weighted κ = 1.0) and fingerprick capillary blood (weighted κ = 0.96) results.

The overall diagnostic accuracy of clinic-based CrAg LFA testing was limited (Table 3). Venous whole blood, fingerprick capillary blood, and urine testing had an overall diagnostic sensitivity of 43% (95% CI 18–71%), 36% (95% CI 13–65%), and 50% (95% CI 23–77%). Sensitivity did not change appreciably when restricted to participants with CD4 < 200 cells/mm^3^ or < 100 cells/mm^3^. Positive predictive values were highest (40%) for venous whole blood CrAg LFA testing among participants with CD4 < 200 cells/mm^3^. Similarly, positive likelihood ratios were 16.6 (95% CI 6.9–39.6) and 9.3 (95% CI 3.3–26.1) for venous whole blood CrAg LFA testing in participants with CD4 < 200 cells/mm^3^ and < 100 cells/mm^3^. CrAg LFA testing on urine samples had low positive predictive values (6–13%) and low positive likelihood ratios (2.2–4.9), regardless of CD4 T-cell count.

**Table 3:** Diagnostic accuracy of a rapid cryptococcal antigen lateral flow assay (CrAg LFA) on venous whole blood, fingerprick capillary blood, and urine for screening HIV-infected South African adults (N = 1,296).

	#TP/(#TP + #FN)	Sensitivity% (95% CI)	#TN/ (#TN + #FP)	Specificity% (95% CI)	PPV% (95% CI)	NPV% (95% CI)	Positive LR(95% CI)	Negative LR(95% CI)
**Clinic-based venous whole blood CrAg LFA**								
All participants	6/14	43 (18, 71)	1127/1149	98 (97, 99)	21 (12, 36)	99 (99, 100)	22.4 (10.8, 46.6)	0.58 (0.37, 0.92)
CD4 < 200 cells/mm^3^	6/13	46 (19, 75)	314/323	97 (95, 99)	40 (22, 61)	98 (96, 99)	16.6 (6.9, 39.6)	0.55 (0.33, 0.92)
CD4 < 100 cells/mm^3^	4/9	44 (14, 79)	140/147	95 (90, 98)	36 (17, 61)	97 (94, 98)	9.3 (3.3, 26.1)	0.58 (0.32, 1.1)
**Clinic-based fingerprick capillary blood CrAg LFA**								
All participants	5/14	36 (13, 65)	1111/1149	97 (95, 98)	12 (6, 22)	99 (98, 99)	10.8 (5.0, 23.3)	0.66 (0.45, 0.98)
CD4 < 200 cells/mm^3^	5/13	38 (14, 68)	311/321	97 (94, 98)	33 (17, 56)	97 (96, 98)	12.4 (4.9, 31.0)	0.64 (0.41, 0.98)
CD4 < 100 cells/mm^3^	3/9	33 (7, 70)	141/147	96 (91, 98)	33 (13, 63)	96 (94, 97)	8.2 (2.4, 27.4)	0.70 (0.44, 1.1)
**Clinic-based urine CrAg LFA**								
All participants	7/14	50 (23, 77)	1021/1137	90 (88, 91)	6 (3, 9)	99 (99, 100)	4.9 (2.8, 8.5)	0.56 (0.33, 0.94)
CD4 < 200 cells/mm^3^	7/13	54 (25, 81)	272/318	86 (81, 89)	13 (8, 21)	98 (96, 99)	3.7 (2.1, 6.6)	0.54 (0.30, 0.97)
CD4 < 100 cells/mm^3^	4/9	44 (14, 79)	115/144	80 (72, 86)	12 (6, 23)	96 (93, 98)	2.2 (0.99, 4.9)	0.70 (0.39, 1.3)

CI = confidence interval; CrAg = cryptococcal antigen; EIA = enzyme immunoassay; FN = false negative; FP = false positive; LFA = lateral flow assay; LR = likelihood ratio; NPV = negative predictive value; PPV = positive predictive value; TN = true negative; TP = true positive.

When applying a combined gold-standard definition of cryptococcal infection being either laboratory-based serum CrAg EIA-positive or positive by latex agglutination testing, an additional eight people were considered to have cryptococcosis (Table 4). Overall, 9 (39%), 6, (26%), and 9 (39%) of the 23 people positive by either laboratory-based serum CrAg or latex agglutination had a positive CrAg LFA test. However, this expanded definition for cryptococcal infection had minimal changes on the diagnostic sensitivity and specificity values of the clinic-based CrAg LFA test for venous whole blood, fingerprick capillary blood, and urine specimens.

**Table 4:** Diagnostic sensitivity and specificity of a rapid cryptococcal antigen lateral flow assay (CrAg LFA), using either positive serum CrAg enzyme immunoassay (EIA) or positive latex agglutination as gold-standard test result.

	#TP/(#TP + #FN)	Sensitivity% (95% CI)	#TN/(#TN + #FP)	Specificity% (95% CI)
**Clinic-based venous whole blood CrAg LFA**				
All participants	9/23	39 (20, 61)	1,121/1,140	98 (97, 99)
CD4 < 200 cells/mm^3^	8/18	44 (22, 69)	311/318	98 (96, 99)
CD4 < 100 cells/mm^3^	5/11	45 (17, 77)	139/145	96 (91, 98)
**Clinic-based fingerprick capillary blood CrAg LFA**				
All participants	6/23	26 (10, 48)	1,103/1,140	97 (96, 98)
CD4 < 200 cells/mm^3^	5/18	28 (10, 53)	306/316	97 (94, 98)
CD4 < 100 cells/mm^3^	3/11	27 (6, 61)	139/145	96 (91, 98)
**Clinic-based urine CrAg LFA**				
All participants	9/23	39 (20, 61)	1,014/1,128	90 (88, 92)
CD4 < 200 cells/mm^3^	8/18	44 (22, 69)	268/313	86 (81, 89)
CD4 < 100 cells/mm^3^	4/11	36 (11, 69)	113/142	80 (72, 86)

CI = confidence interval; CrAg = cryptococcal antigen; EIA = enzyme immunoassay; FN = false negative; FP = false positive; LFA = lateral flow assay; TN = true negative; TP = true positive.

When evaluating results by grade, tests with a high CrAg LFA grade (5+) were more likely to be laboratory-based serum CrAg-positive for venous whole blood (4/11; 36%), as compared to tests with low CrAg LFA grade (1+) (2/15; 13%). Similar results were observed for fingerprick capillary blood tests [3/9 (33%) for high grade versus 1/26 (4%) for low grade], and for urine tests [1/1 (100%) for high grade versus 3/98 (3%) for low grade.

**Figure 2:**
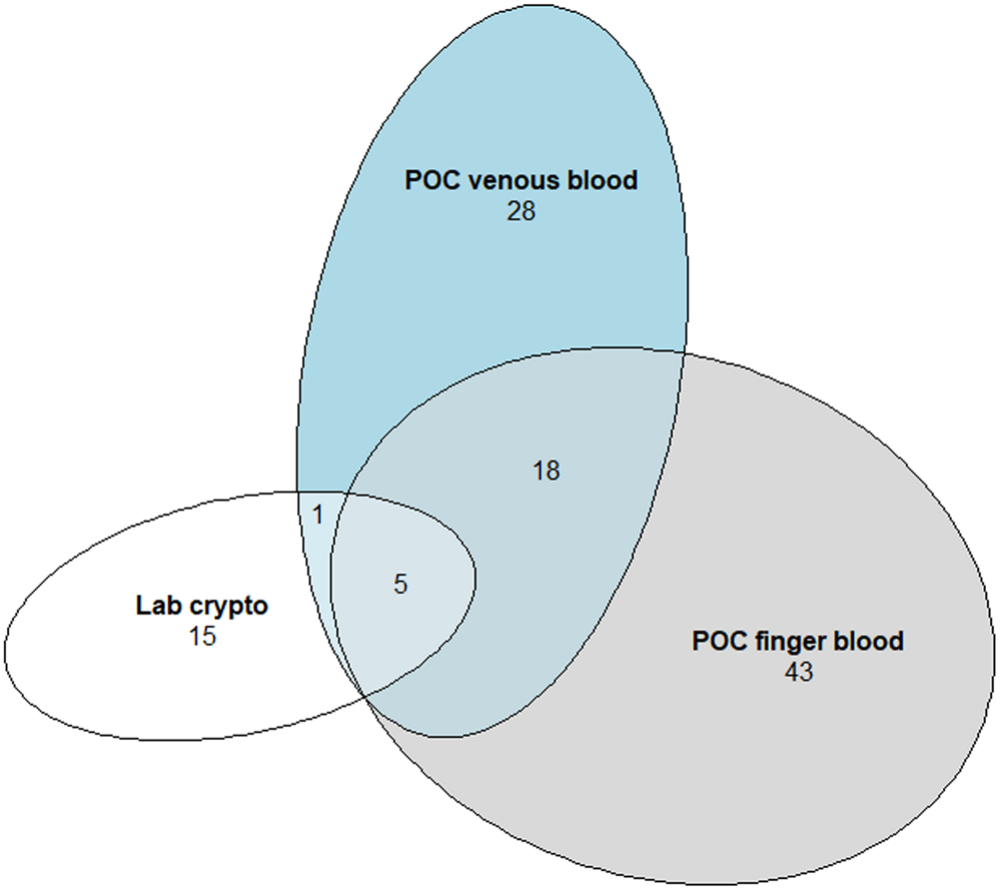
Venn Diagram for agreement between all positive laboratory-based serum CrAg EIA results (“Lab crypto”) and point-of-care (POC) CrAg by venous and fingerprick whole blood.

The overlap for test results is shown in Fig. 2. Among tests that were considered to be false positives, by comparison to laboratory-based CrAg EIA testing, the CD4 count medians were 258 cells/mm^3^ [interquartile range, IQR 87, 399], 299 cells/mm^3^ [145, 606], and 250 cells/mm^3^ [96,400] for venous whole blood, fingerprick capillary blood, and urine testing, respectively.

In additional post hoc testing of 14 thawed serum CrAg EIA-positive samples, 13 samples were serum CrAg LFA-positive, and one sample was serum CrAg LFA-negative. All 29 randomly selected CrAg EIA-negative samples were also negative by serum CrAg LFA testing. Thus, when tested on thawed serum samples, the CrAg LFA test had a diagnostic sensitivity of 93% (95% CI 66–100%) and specificity of 100% (95% CI 88–100%), which conferred to high positive and negative predictive values.

In the post hoc hospital sub-study of 30 adults with a diagnosis of HIV-associated cryptococcal meningitis, the mean age was 34.6 (SD ± 10.1) years and 8 (26.7%) participants were female. The median CD4 count was 27 cells/mm^3^ (interquartile range: 7–53 cells/mm^3^), 27 (90%) participants had CD4 < 200 cells/mm^3^, and 19 (63%) had initiated ART. Within this cohort, all participants had a positive serum CrAg LFA assay. All participants were also CrAg LFA-positive by both venous whole blood and fingerprick capillary blood, while 28 of 30 (93%) were CrAg LFA test positive by urine specimen (Table 5). The grade for the positive test results were higher for venous whole blood and fingerprick capillary blood, as compared to urine specimens.

**Table 5:** Distribution of results and test scores from hospital-based cryptococcal antigen lateral flow assay (CrAg LFA) testing of participants with HIV-associated cryptococcal meningitis.

CrAg LFA test	Venous whole blood CrAg LFA	Fingerprick capillary blood CrAg LFA	Urine CrAg LFA
Negative	0	0	2
Positive	30	30	28
**Total**	**30**	**30**	**30**
**Grade for Positive Tests**			
1 + (low)	2	3	11
2+	5	4	12
3+	5	9	5
4+	10	7	0
5 + (high)	8	7	0
Mean ± SD	3.6 ± 1.2	3.4 ± 1.3	1.8 ± 0.7

CrAg = cryptococcal antigen; LFA = lateral flow assay; SD = standard deviation.

## Discussion

In this cohort of HIV-infected ambulatory adults in Durban, South Africa, a rapid CrAg LFA test was feasible when performed at the clinical point of care by trained nurses. The CrAg LFA had limited diagnostic accuracy for use on venous whole blood and fingerprick capillary blood, which was likely due to lower circulating CrAg titers in these specimens compared to serum. As has been shown in other studies, the CrAg LFA perform well on serum specimens and among participants with cryptococcal meningitis with high circulating titers of CrAg. Despite some limitations, clinic-based CrAg LFA screening for HIV-infected adults with participants with CD4 < 200 cells/mm^3^ may be an important tool to help accelerate diagnosis and treatment of HIV-associated cryptococcal infections in order to reduce HIV-associated cryptococcal mortality.

The estimated prevalence of cryptococcal antigenemia in our cohort was consistent with other studies of HIV-infected adults in sub-Saharan Africa^1,19^. An older study in Cape Town reported a higher CrAg prevalence of 12% (42/336) among pre-ART HIV-infected adults with CD4 ≤100 cells/mm^3^ ^5^, and the overall incidence of HIV-associated cryptococcosis has been declining throughout South Africa since the year 2006^27^. In our cohort, 4 of 15 (27%) people with cryptococcal antigenemia had a CD4 count of 100–199 cells/mm^3^, which provides further support for a CD4 threshold of 200 cells/mm^3^ for universal CrAg screening^11^. In addition, CrAg screening should be applied to both ART-naïve and ART-experienced HIV-infected people^20^.

In multiple studies, the rapid CrAg LFA test has proven accurate when compared to CrAg EIA and latex agglutination testing in serum and/or cerebrospinal specimens^13–16,21,22^. However, one study has reported false negative results from CrAg LFA testing on serum due to the presence of a prozone^23^. Cryptococcal antigens circulate at lower levels in urine than in serum^13^, and two other studies have demonstrated poor sensitivity of the CrAg LFA for use with urine specimens^24,25^. One Ugandan study reported good accuracy of the CrAg LFA when used on fingerprick capillary blood from HIV-infected adults with cryptococcal meningitis^22^, which is also consistent with our findings. A newer semi-quantitative rapid CrAg lateral flow assay, Biosynex^®^ CryptoPS (Biosynex Diagnostics, Strasbourg, France) was shown to have good accuracy when used on serum and may indicate the presence of cryptococcal meningitis^26^.

We validated the CrAg LFA test on venous whole blood, fingerprick capillary blood, and urine specimens, since they can be easily obtained in clinics and tested at the clinical point of care. To our knowledge, the CrAg LFA had not been previously validated as a clinic-based screening test on venous whole blood or fingerprick capillary blood by trained nurses. We performed rapid CrAg LFA testing on fresh, not frozen, samples, as would be practiced in ambulatory clinics, and used a CrAg EIA test as the gold standard that was a direct comparator to the rapid CrAg LFA. The relatively fewer participants with cryptococcal antigenemia and/or CD4 immunosuppression reflected real-world practice in South Africa^27^, but was a limitation for diagnostic accuracy measurements. In this study, we did not reproduce the CrAg LFA test limit of detection that had been reported by the test manufacturer, or evaluate for non-specific binding of the CrAg LFA.

Overall, the limited diagnostic performance may have been a result in part due to lower levels of circulating cryptococcal antigens in venous whole blood and fingerprick capillary blood among ambulatory patients. The false positive CrAg LFA test results may be related to non-specific binding of a similar antigenic epitope that may be removed for analyses with serum, and this interference might be overcome higher circulating CrAg titers who more likely to have cryptococcal meningitis^28^. However, the CrAg LFA test detects ambulatory patients with high circulating blood CrAg titers or subclinical cryptococcal meningitis. A recent report from South Africa found no reduction in annual case fatality ratio for cryptococcal meningitis, which was attributed to delays in diagnosing HIV-associated cryptococcal infections^27^. In the REALITY trial, relative clinical benefits of fluconazole prophylaxis were observed for both CrAg-positive and -negative patients, which may have been due in part to imperfect CrAg test sensitivity^9^. The absolute benefits were still greater for CrAg-positive patients, and the authors concluded that CrAg screening should be routine for HIV-infected adults with CD4 < 100 cells/mm^3^ ^9^.

In conclusion, rapid CrAg LFA screening was feasible when performed by trained nurses at the clinical point of care, and had limited sensitivity on venous and fingerprick blood likely due to lower circulating CrAg titers. Additional clinic-based studies are needed to determine whether CrAg LFA screening among HIV-infected adults at the clinical point of care may identify a sufficient number of infected persons, reduce treatment delays, and lead to improved patient outcomes. The rapid CrAg LFA is a simple and inexpensive tool that if used among immunosuppressed HIV-infected persons could help decrease the large burden of AIDS-related deaths from cryptococcal meningitis in sub-Saharan Africa and worldwide.
